# Supplementary figures and images for: Topoisomerase IIα prevents ultrafine anaphase bridges by two mechanisms
Source: Open Biol. 2020 May 13;10(5):190259. doi: 10.1098/rsob.190259 (PMC7276528; doi:10.1098/rsob.190259)

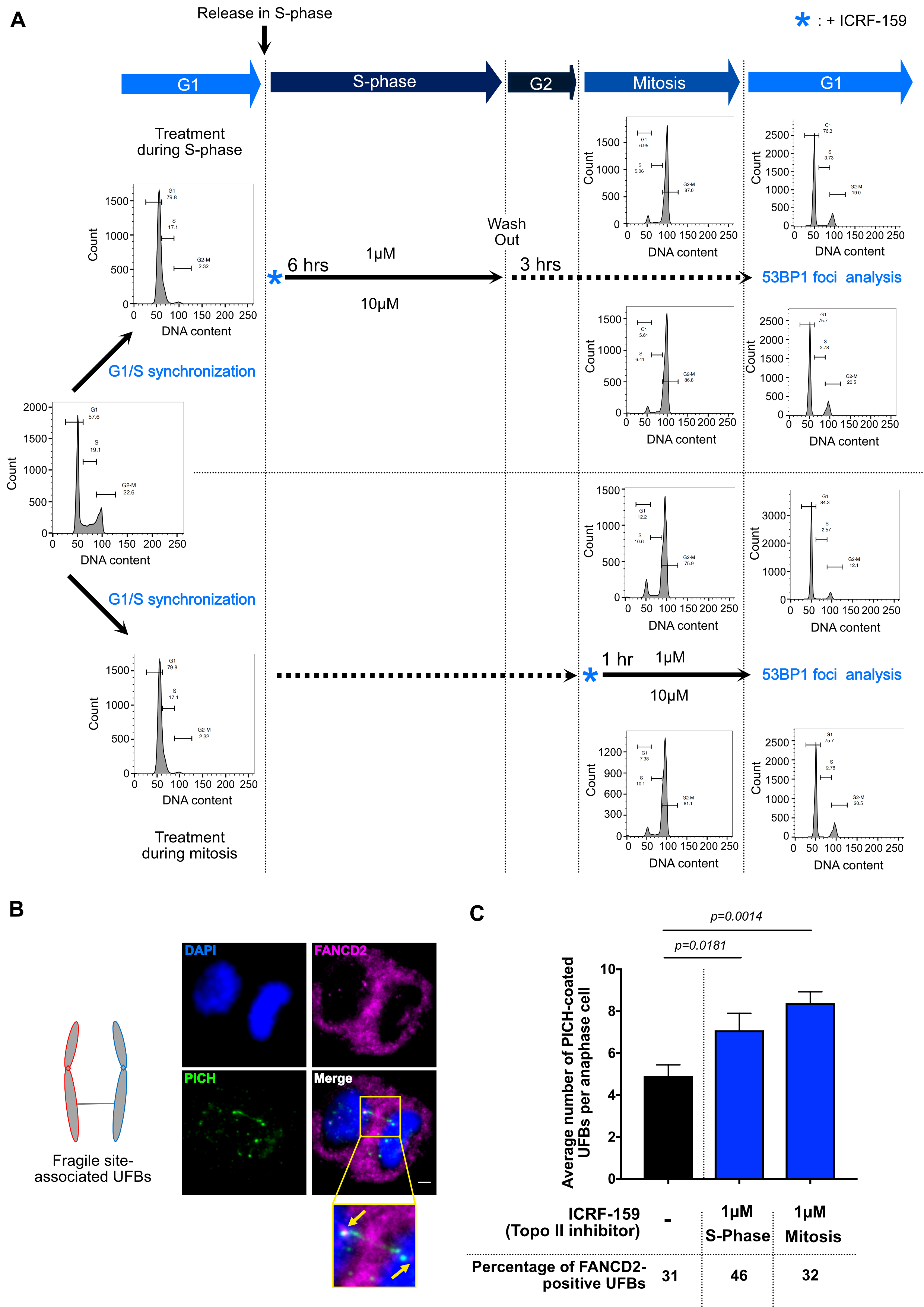

Supplement: Figure S1: Topo IIα inhibition during mitosis promotes centromeric-associated UFB formation [file rsob190259supp1.tiff]

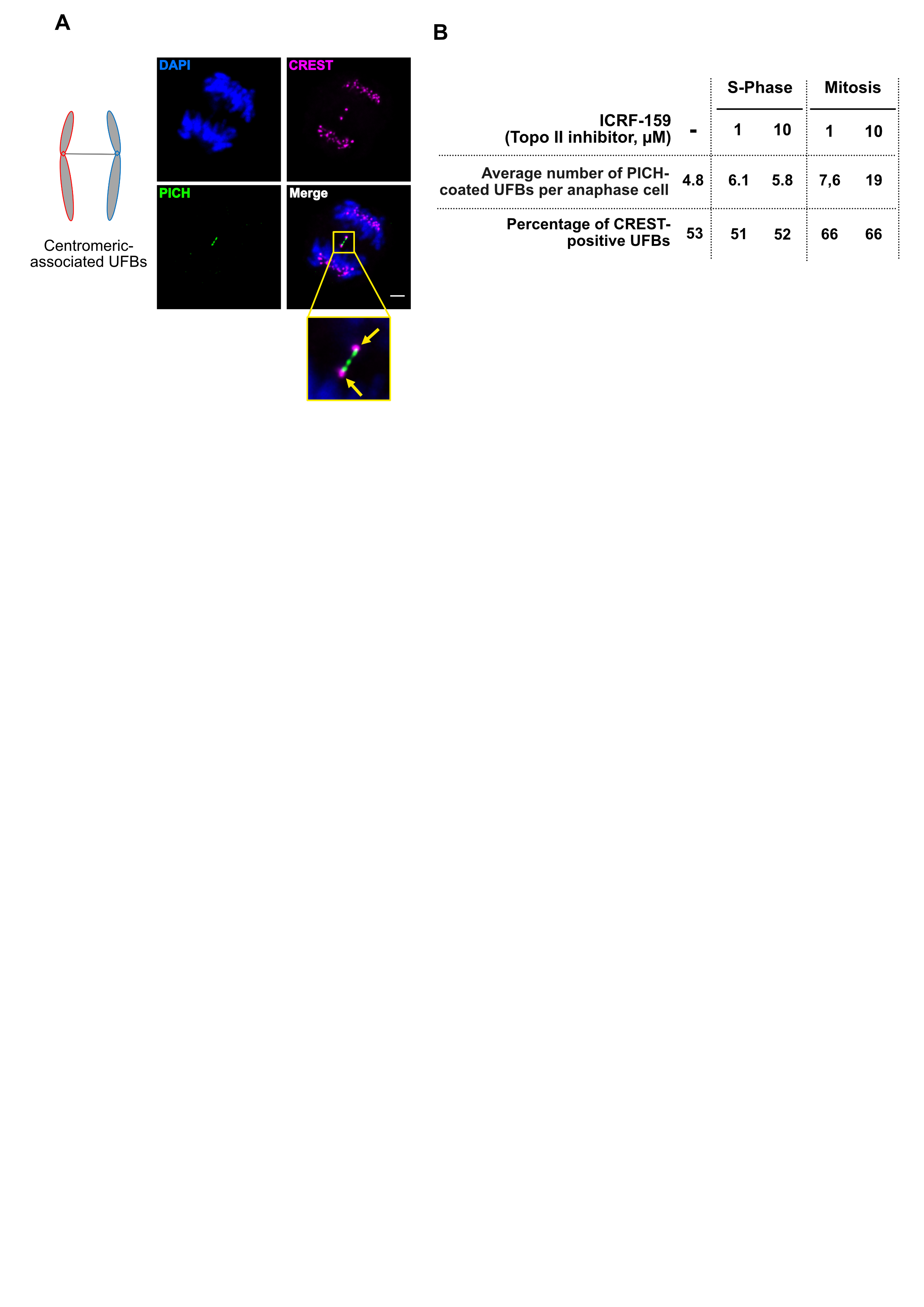

Supplement: Figure S2: Cell cycle synchronization does not affect UFB formation upon Topo IIα inhibition [file rsob190259supp2.tiff]
